# Extended parallel process model (EPPM) in evaluating lung Cancer risk perception among older smokers

**DOI:** 10.1186/s12889-021-11896-1

**Published:** 2021-10-17

**Authors:** Fatemeh Zarghami, Hamid Allahverdipour, Mohammad Asghari Jafarabadi

**Affiliations:** 1 National Elites Foundation, Center For International Science and Technology Cooperation (CISTC), Tehran, Iran; 2 Department of Gerontology, Faculty of Health, Tabriz University of Medical Sciences, Tabriz, Iran; 3 Virginia Polytechnic Institute and State University, Department of Population Health Sciences & Center for Gerontology, Blacksburg, Virginia USA; 4 Department of Health Education and Health Promotion, Faculty of Health, Tabriz University of Medical Sciences, Tabriz, Iran; 5 Department of Statistics and Epidemiology, School of Medicine, Zanjan University of Medical Sciences, Zanjan, Iran; 6 Center for the Development of Interdisciplinary Research in Islamic Sciences and Health Sciences, Tabriz University of Medical Sciences, Tabriz, Iran

**Keywords:** Risk perception, Lung cancer, Conceptual model, Smoking, High readiness, Older smoker

## Abstract

**Background:**

There is a lack of scientific literature on the application of fear appeals theories to evaluate lung cancer risk perception among smokers. The aim of the present study is to apply the Extended Parallel Process Model (EPPM) to discover the perception of the smokers about their lifetime risk of developing lung cancer (perceived susceptibility), their perception of lung cancer survival (perceived severity), response efficacy, self-efficacy, and readiness to quit.

**Methods:**

In this cross-sectional study, 215 eligible smokers (aged 45 years and over who have smoked at least 1 pack per day in the last 5 years) were recruited. The data collection tool was designed using validate self-report questionnaires and it was contained items on the perceived risk of a smoker contracting lung cancer and perceived lung cancer survival rate. It also had questions to measure the main constructs of the EPPM and Readiness to quit (“Low_Readiness”, and “High_Readiness”). To test how the data support conceptual EPPM to data, Generalized Structural Equation Modeling (GSEM) was used.

**Results:**

Findings showed a significant relationship between Perceived_Susceptibility and Perceived_Response Efficacy; (B = 1.16, *P* < 0.001); between Perceived_Susceptibility and Perceived_Self Efficacy, (B = -0.93, P < 0.001), Perceived_Severity, and Perceived_Response Efficacy (B = 1.07, P < 0.001). There was also a significant relationship between Perceived_Threat and Perceived_Response Efficacy; between Perceived_Threat and Perceived_Self Efficacy. The relationship between High_Readiness and Perceived_Self Efficacy, and between High_Readiness and Perceived_Severity also were significant. However, the relationships between High_Readiness and Perceived_Threat were not significant (*P* > 0.05).

**Conclusion:**

Perceived_threat and Perceived_efficacy were important for smokers with low readiness to quit, while Perceived_efficacy was most important for smokers with high readiness to quit. These findings could be used in promoting lung cancer awareness and designing smoking cessation programs based on smokers’ stages of change.

## Background

Recent estimates show that almost one-third of the world population smokes [1]. Smoking imposes a significant economic burden on the health system and society as a whole [2]. Besides, the largest proportion of cancer death is related to lung cancer [2]. According to the results of a review article by Moosazadeh et al., one-fifth of Iranian men and around 2–3% of women smoke on an everyday basis and this pattern even increases significantly after the age of 30 [3]. It is estimated that 55% of lung cancer deaths in women and over 70% of lung cancer deaths in men are due to smoking [1].

The second most common cancer in both men and women is lung cancer which is one of the deadliest cancer types around the world [4]. There have been advances in lung cancer treatment in the past decades; however, the 5-year survival rate (2009–2015) remains very low at only 19.4% [5]. Lung cancer is the leading cause of cancer death globally [6]. Active cigarette smoking is the main cause of lung cancer [7, 8]. People who smoke cigarettes are 15 to 30 times more likely to develop lung cancer or die from lung cancer [9]. Medical research has confirmed that smoking leads to lung cancer [7] and tobacco use is the leading cause of lung cancer [1]. Inconsistent with tobacco control, numerous activities performed at the local, national, and international levels are emphasizing behavior change by using health communication strategies and behavior change theories [10]. Effective risk communication depends on presenting general risk factors and preventive information, and also on factors that are specific to the individual [10]. Behavior models such as the Health Belief Model and Self-Regulation Model, have been used to investigate how smokers perceive the risk of lung cancer [11]. There are also studies such as the study conducted by Chen et al. that postulates emotion-based (or parallel processing) theories that discuss pathways shaping risk perceptions [12].

### Extended parallel process model

One of the fear appeal theories is the Extended Parallel Process Model (EPPM), which incorporates effective processes (i.e., fear) in risk communication [13]. This study is designed to apply EPPM in evaluating the risk of acceptance in the context of personalized risk communication and effects of the intervention on intention to quit among smokers [13]. Kim Witte explains what leads to danger control versus fear control [13]. Then, she identified the underlying mechanisms occurring in each process [11]. Prior cancer-related studies have examined the EPPM in relation to smoking cessation [14, 15], mammography [16], and skin cancer screening [17].

The proposed model of the study postulates that when an individual is conferred with a fear-arousing message, two cognitive appraisal processes will begin: (a) threat appraisal and (b) efficacy appraisal. In the threat appraisal, the individual examines two characteristics of the perceived threat which are severity and susceptibility. Severity appraisals entail finding out how much harm can be caused by the threat (e.g., ‘Lung Cancer can kill me.’), while susceptibility appraisal is about the estimation of possibility of the threat is affecting the individual (e.g. ‘Because I am a smoker, I will develop lung cancer in my lifetime.’). If the perceived threat is considered to be, low, it is unlikely that the individual will process the message any further. However, if the perceived threat is high, the individual will enter the efficacy appraisal phase.

In response, the efficacy of the individual determines how much the recommended behavior is effective in avoiding the threat. (‘Quitting smoking can lower my chance of developing lung cancer.’). In self-efficacy, the individuals evaluate their capability to achieve the recommended behavior (‘I think I am capable to stop smoking.’). When both threat and efficacy appraisals are high, the individual will enter a cognitive process with the goal of controlling the danger, and therefore, they will engage in adaptive behavior (e.g., taking steps to quit smoking). However, If threat appraisals are high but efficacy is low, the individual will start a cognitive process to control the fear rather than the danger itself. This process will lead to dysfunctional reactions such as defensive avoidance (e.g., ‘I will not think about that!’) [18].

### Lung cancer risk perception

One of the factors that affect decision-making based on the risk perception, is an individual’s frame of reference developed over a lifetime [19, 20]. Other researchers have used the EPPM in relation to various cancerous diseases such as breast cancer [21–24], skin cancer [25, 26], and colorectal cancer [18, 27, 28].

For instance, findings of the study conducted by Birmingham et al. provided support of the utility of Extended Parallel Process Model to motivate colorectal cancer screening in persons at increased risk [18]. Results of the literature review identified that there is no study (if any) to determine the effectiveness of EPPM to evaluate the risk perception of lung cancer among smokers. Therefore this study aimed to apply EPPM in evaluating the perception of the smokers about their lifetime chance of developing lung cancer and their perception about lung cancer survival.

## Methods

### Participants and procedure

This project is a cross-sectional study with a survey as a collection tool and a sample size of 215 individuals between April 2019–July 2019 in Tabriz, Iran. Participants were smokers age 45 or older who had smoked at least 1 pack per day (or more) in the past 5 years. Interviewers read the questions to the participants and their responses were collected by the interviewers. The survey questions were adapted from validated surveys (English) to determine the effectiveness of the EPPM model in evaluating smokers’ perception of lung cancer risk [18, 29–31]. This process was performed after the forward-backward translation of the survey and checking for their reliability and validity, and cultural adaptation of the questionnaire. The final survey (Farsi) was distributed among eligible participants in locations such as parks, bus stations, retirement houses, senior health organizations, Clinic of pulmonary disease in Imam Reza hospital, and Razi hospital in Tabriz city. Iran’s National Elite Foundation, Center For International Science and Technology Cooperation (CISTC) approved the initial proposal of this cross-sectional study. All methods were carried out in accordance with relevant guidelines and regulations of Tabriz University of Medical Sciences. The Institutional Review Board (IRB) of this university approved the study and granted the IRB code (IR.TBZMED.REC.1397.955).

### Study size

A sample size of 138 achieves 95% power to detect a correlation of 0.3 between the main outcomes of the study including lung cancer risk perception and self-efficacy (as found in Birmingham et al., 2015) using a two-sided hypothesis test with a significance level of 0.05. Taking into account, the design effect of the sampling procedure equals 1.5, the final sample size increased to 207 cases [32–34]. Finally, a sample size of 215 participants were recruited for this study.

In the factor analysis, a sample must include at least five subjects per item [35]. Considering a total number of 28 items, a sample size of 140 was required which our sample size of 215 fulfills the minimum requires sample size.

### EPPM theoretical constructs

A) Perceived self-efficacy: There are 4 questions designed to measure this variable and are measured in three categories of “yes”, “no”, and “maybe”. These questions are: 1) *I feel that I have little control over the smoking associate risk on my health. 2) If I get complications from lung cancer, there is not much that I can do about it. 3) My own efforts can help control my risk of developing lung cancer complications. 4) If I make good efforts to control lung cancer, I am less likely to get serious complications.*
B) Perceived response-efficacy: There are 2 questions to measure this variable which are in three categories of “yes”, “no”, and “maybe” and are asking about the effect of smoking cessation on the likelihood of contracting lung cancer and survival of lung cancer. These questions are: 1) *If I quit smoking, I am less likely to develop lung cancer. 2) If I get lung cancer in my lifetime, quitting smoking is effective to prevent complications.*
C) Perceived susceptibility: There are 3 questions designed to measure this variable. Two of these questions are on the scale of “0” to “10” and one question was about the perceived risk of developing lung cancer in comparison to the individuals of the same age and sex category. These three questions are: 1) *If you continue to smoke, what is your chance of developing lung cancer? Please choose a number from “0” to “10”, with “0” as having zero chance of developing lung cancer and “10” as having a definite chance of lung cancer. 2) If you continue smoking, what do you think about your risk of developing lung cancer compared to other people of the same age and sex. Answer choices are 1: “less chance”, 2: “more chance”, and 3: “equal chance”. 3) Because I am a smoker, I am more likely to develop lung cancer compared to other people of the same age and sex. Answer choices are “yes”, “no”, and “maybe”.*
D) Perceived severity: Two questions are designed to measure this variable. One of these questions is on the scale of “0” to “10” and the second question is asking about perceived lung cancer survival in comparison to the individuals of the same age and sex category. These questions are: 1) *If you continue to smoke and develop lung cancer, what is your chance of being alive after 5 years? Please choose a number from “0” to “10”, with “0” as having zero chance of surviving the lung cancer and “10” as having a definite chance of surviving the lung cancer (definite survival). 2) Because I am a smoker, I am more likely to have serious lung cancer complications compare to other people of the same age and sex. Answer choices are “yes”, “no”, and “maybe”.*
E) Perceived fear/threat: There are 2 questions that measure the fear and worry of the participants of developing lung cancer and lung cancer survival. These 2 questions are: 1) *Because I am a smoker, I am very concerned to develop lung cancer. 2) Because I am a smoker, I am very concerned to have serious lung cancer complications.*

## Measures

Levels of cigarette consumption, such as the number of cigarettes smoked per day, the number of days smoked per month, and the amount of lifetime cigarette use has been used differently by researchers around the world to define smoking status [36]. The risk of lung cancer drops substantially (39.1%) among those who were heavy smokers in the past and who had a history of quitting compared to current smokers, and this lower risk was detectable within 5 years of quitting [37]. Therefore, in our study, smokers are defined as individuals who smoked at least 1 pack per day in the past 5 years. The definition of “heavy smoker” is varied in the literature. However, based on most definitions smoking more than 1 pack per day is considered “heavy smoking” [38]. We recruited heavy smokers to obtain more understanding of their perception about the effects of smoking on lung cancer; considering the fact that smoking is the major preventable risk factor for lung cancer advertised in public health education programs for smoking cessation. Most of the diagnosed cases of lung cancer are 65 or older and only a very small number of people are diagnosed with lung cancer younger than the age of 45 [39]. This means the chance of developing lung cancer under the age of 45 is very small [40]. Therefore, in this study the age 45 and over, has been chosen as age inclusion criteria.

In this process, the data collection tool was designed using validated questionnaires both in Persian and English language, and it was contained questions on the perception of getting lung cancer and its survival, and questions based on the constructs in the EPPM model. Highly experienced lung cancer and health education experts (10 individuals) reviewed the final Persian version of the questionnaire and confirmed its content and face validity in quantitative and qualitative manners. Their comments were later reviewed by the research team and the necessary modifications were applied [31].

### Background characteristics

Demographic characteristics of the participants have been measured with the checklist designed from the validated surveys found in the literature, with cultural adaptation. These variables include Sex, Ethnicity (Fars, Azeri, Other), Marital status (Engaged, Single, Married, Divorced), Age (years), Education (Middle school, High School, Diploma, Associate degree, Master, Doctorate), Occupation (Farmer, Teacher, Driver, Worker, Office Employee, etc) and Income (<IRR 500,000, IRR 500,00-IRR1000,000, up to >IRR4,000,000). Three variables, 1.Education 2.Occupation 3.Income were used to create a new variable “SES.score” (Score for Socio-Economic Status). In this process, first, each of the variables Education, Occupation, and Income was transformed on a scale of 1–6. For instance, variables “Occupation”, the “Office employee” and “Receptionist” were given the same score, and “University faculty” and “Teacher” were also given the same score (value, here). Finally, a combination of these scores for three variables (Education, Occupation, and Income) was used to create a variable of SES and then the “SES.level” was categorized on the scale of 1–3 (Low, Medium, and High).

**Fig. 1:**
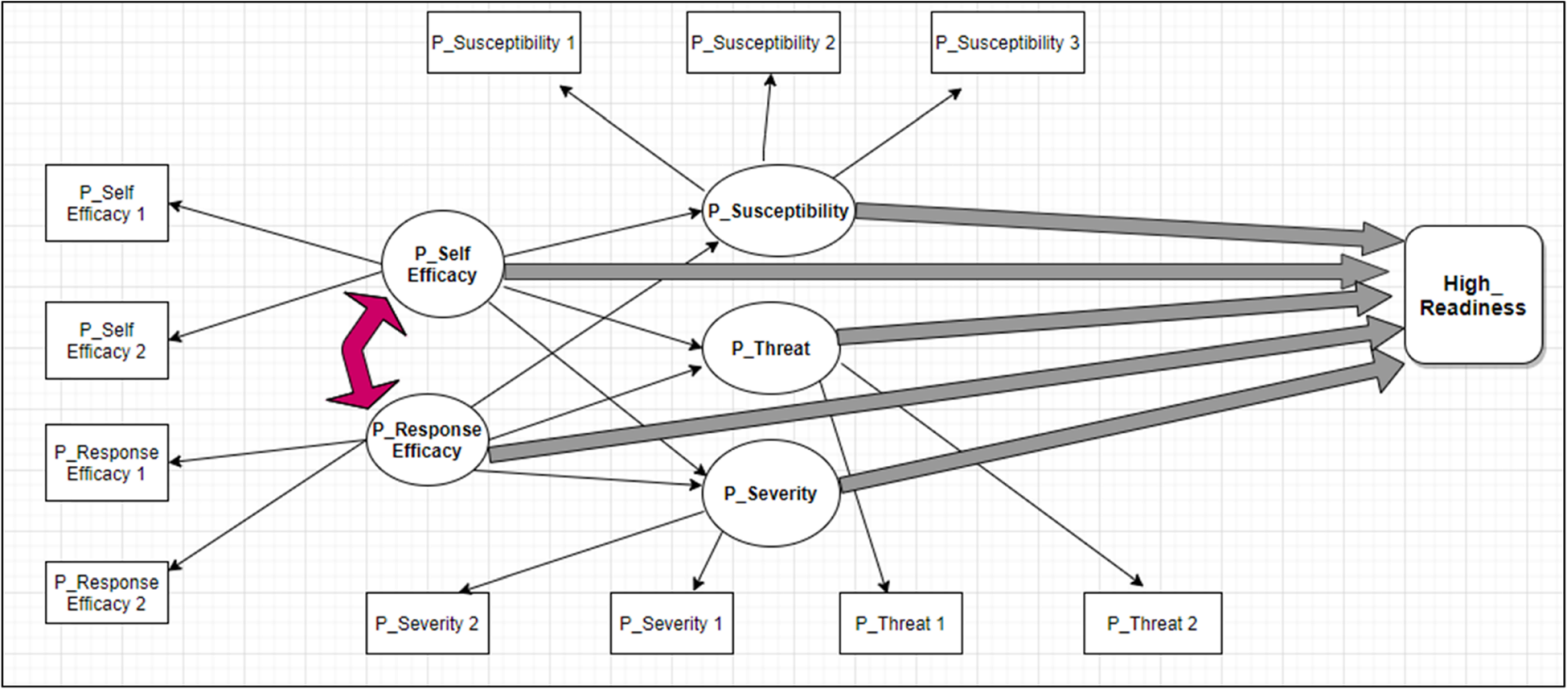
Initial Conceptual Model. (*P: Perceived)

There were two groups of constructs in the Structural Equation Modelling (SEM) [1]: EPPM constructs (Perceived self-efficacy, Perceived response-efficacy, Perceived susceptibility, Perceived severity, Perceived fear/threat), and [2] Main outcome measures (Readiness to quit, High_Readiness and Low_Readiness). An initial conceptual framework was developed for this study based on the original EPPM model explained by Kim Witte and another model is discussed in the article by Birmingham et al. [18]. This model and the relationship between the constructs have been shown in Fig. 1:

Two constructs of the EPPM (Perceived susceptibility, and Perceived severity) have been used as the measure for lung cancer risk perception. Lung cancer risk perception was first measured in two separate categories. The first category measures “Perceived susceptibility” and is on a scale of “0” to “10”. The next variable is measuring “Perceived severity” and is also on a scale of “0” to “10” [12]. Based on the conceptual model and our measures of the construct, we add “Perceived_Susceptibility” latent variable with three indicators, and “Perceived_Self Efficacy” latent variable with two indicators. Details of these indicators are in Fig. 1.

### Main outcome: low and high readiness to quit

When individuals are in the protection motivation process of the EPPM, they have high readiness to quit and when they are in the defensive motivation of the model, they have “low readiness” to quit. Readiness to quit was defined as early and later stages of change, consecutively. The questions that are designed to measure the stages of change are listed below:

### Are you seriously considering quitting smoking within the next 6 months?

1. *Are you planning to quit in the next 30 days?*
2. *If yes, are you currently in the status of quitting smoking?*
3. *If yes, how long have you been in the quitting smoking status?*
4. *What were your reasons for quitting smoking in the past? (Open-ended question)*

The concept of “stage of change” has been widely accepted in the literature and it has commonly been used for behavioral modification in areas such as smoking cessation, dieting, regular exercise, and seatbelt use [41]. Individuals progress through a series of stages in smoking cessation: recognizing the need to change, contemplating a change, making a change, and finally sustaining the new behavior [42] (Precontemplation, contemplation, preparation, action, and maintenance). The first 3 questions are designed in the categories of “yes”, “no”. For the 4th question, the answer choices are 1) 1 week, 2) 1 week to 1 month, 3) 1 month to 2 months, 4) More than 3 months, and the last question is open-ended. It is possible to determine the stage of change of participants by these questions. The responses to these 3 questions are transformed into new categories to determine the final stage of change of the participants. Individuals who were identified in the stages of “Precontemplation” and “contemplation” was labeled as individuals with “Low_Readiness” and individuals who were in the stage of “preparation”, “action”, and “maintenance” were labeled as “High_ Readiness”.

### Statistical analysis

Statistical analyses were performed using MPlus (7.4) [43] and SPSS [17] (SPSS Inc., Chicago, IL, USA). To test the fitness of the measurement model and to fit the conceptual EPPM to data, generalized Structural Equation Modeling was used [43]. To investigate the fitness, the goodness of fit indices were calculated. Values smaller than 0.08 for Root Mean Square Error of Approximation (RMSEA), the normed chi2 (chi2 divided by the degrees of freedom) < 5, and values greater than 0.90 for Tucker-Lewis Index (TLI) and Comparative Fit Index (CFI) confirmed the fitness of model [35]. We used a 6 step process in the modeling: model specification based on conceptual theoric model, model identification, model estimation utilizing maximum likelihood method, model testing utilizing the goodness of fit indices and significance of the parameters, model modification utilizing modification indices, and finally model validation.

## Results

### Recruitment

Approximately 500 individuals were approached to assess their eligibility to be part of this study. From this number, 260 were eligible to enter the study. Some participants didn’t consent after explaining the study goals. This was more common among female participants and their concern about their privacy. Finally, a total number of 215 participants were recruited in this study which 192 (89.7%) were current smokers and 188 (89.1%) had smoked 1 pack per day in the past 5 years (or more than 5 years) but they were in the quitting status at the time of the participation.

### Participants profile

The number of male participants was 185 (86.0%) of 215 participants. Nearly a quarter of the participants (*n* = 55; 25.6%) were from low SES levels, more than half of them belonged to the middle SES levels (*n* = 124; 57.7%) and only 36 individuals (16.7%) had a high SES level. The majority of the participants (*n* = 188; 87.4%) were married or engaged. About 20% of the respondents were in the age category of 51–55 and nearly half of the respondents were above age 55 (46.6%). For more information about the demographic characteristics of the participants, please refer to Table 1.

**Table 1:** Demographic charactertistics and SES level of participants

	Frequency	Valid Percent (%)
*SES Level*		
Low	55	25.6
Medium	124	57.7
High	36	16.7
*Marital Status*		
Single	8	3.7
Married-Engaged	188	87.4
Divorced	7	3.3
Widow	12	5.6
*Age*		
45–50 years	72	33.5
51–55 years	43	20.0
56–60 years	38	17.7
61–65 years	29	13.5
66–70 years	10	4.7
71–75 years	14	6.5
> 75 years	9	4.2
*Education*		
Illiterate	21	9.8
End of Elemntary school	51	23.7
End of Middle school	46	21.4
End of High school	42	19.5
End of Associate degree	37	17.2
End of Bachelor’s degree	15	7.0
End of Master’s degree	1	.5
Ph.d. and more	2	.9
*Occupation*		
Farmer	12	5.6
Factory worker	9	4.2
Receptionist	1	.5
Teacher	5	2.3
University faculty	3	1.4
Driver	49	22.8
Office employee	81	37.7
Home keeper	21	9.8
Other	34	15.8
*Recruitment Location*		
Parks	45	20.9
Bus stations	29	13.4
Retirement houses	24	11.1
Senior organizations	18	8.3
Imam Reza hospital	25	11.6
Razi hospital	50	23.2
Other	24	11.1

### Smoking history and quitting intention

Years of smoked among participants had a minimum-maximum of 1–68 years with a mean of 30.8 (SD = 13.1). Most of the participants (199) had a smoking history of 1–2 packs per day and only 16 of them smoked 2–4 packs per day. Participants with quitting attempts in the past identified their reasons to quit as 1) Diagnosis of chronic disease (e.g. heart), 2) Maintaining overall health, 3) Respecting family members or non-smokers, 4) Cigarettes’ bad smell and 5) Reducing financial burden of cigarettes. This group also believed that their past quitting failures were caused by 1) Lack of motivation and entertainment, 2) Financial, work, and family problems, 3) Influence of smoker friends/partner and 4) Stress and anxiety. Participants’ readiness to quit is measured as having low and high readiness to quit. Of 215 participants, 147 individuals (68.37%) were in the group of Low_Readiness and 68 of them (31.62%) were in the group of High_Readiness.

### Results of structural equation modeling for EPPM constructs

The bivariate associations among EPPM concepts are not relevant, also because these concepts are seen as theoretically distinct concepts. Theoretically, some correlation is expected but the EPPM is based on the assumption that its concepts are independently associated with intention/readiness. We fit a model with all demographic variables that have a path to “High Readiness”, but since there was no significant relationship between the demographic variable and “High Readiness”, therefore, in the next step we removed these variables from the model for model parsimony. The strongest associations with readiness are expected for efficacy perceptions over and beyond threat perceptions. The full model fitted the data well after some modifications; χ2 (60) = 64.67, *P* = 0.317, > 0.05, χ2/df = 1.08 < 5, TLI = 0.954 > 0.9, CFI = 0.964, > 0.9, RMSEA = 0.019 < .08, (90% CI = (0.001 to 0.047). In fact, the model exactly fits the original EPPM conceptual model which is introduced in the introduction section. In addition, all the relationships between the items and the scales were significant (*P* < 0.05). This applied to all the scales: Perceived_Susceptibility, Perceived_Severity, Perceived_Self Efficacy, Perceived_Response Efficacy, and Perceived_Threat. For more information please refer to Table 2.

**Table 2:** EPPM STANDARDIZED MODEL PARAMETERS

	B (SE)	***P***-Value
^a^**P_Susceptibility BY**		
P_Susceptibility1	26.010 (SE: 3.295)	< 0.000
P_Susceptibility2	3.285 (SE: 1.541)	0.033
P_Susceptibility3	33.869 (SE: 5.373)	0.000
**P_Self Efficacy BY**		
P_Self Efficacy1	18.725 (SE: 5.468)	0.001
P_Self Efficacy 2	19.095 (SE: 4.696)	< 0.001
**P_Response Efficacy BY**		
P_Response Efficacy 1	30.343 (SE: 4.198)	< 0.001
P_Response Efficacy 2	21.076 (SE: 4.113)	< 0.001
**P_SEVerity BY**		
P_Severity1	0.760 (SE: 0.213)	< 0.001
P_Severity2	28.205 (SE:3.623)	< 0.001
**P_Threat BY**		
P_Threat 1	35.349 (SE: 8.800)	< 0.001
P_Threat 2	22.136 (SE: 5.721)	< 0.001
**P_SUSceptibility ON**		
P_Response Efficacy	1.167 (SE: 0.150)	< 0.001
P_Self Efficacy	−0.935 (SE: 0.170)	< 0.001
**P_Severity ON**		
P_Response Efficacy	1.076 (SE: 0.097)	< 0.001
P_Self Efficacy	−0.783 (SE: 0.107)	< 0.001
**P_Threat ON**		
P_Response Efficacy	0.858 (SE: 0.146)	< 0.001
P_Self Efficacy	−0.484 (SE: 0.106)	< 0.001
**HIGH_Readiness ON**		
P_Threat	−0.243 (SE: 0.321)	0.451
P_Self Efficacy	− 22.228 (SE: 3.973)	0.000
P_Susceptibility	0.509 (SE: 0.501)	0.310
P_Severity	−28.240 (SE: 3.655)	< 0.001
P_Response Efficacy	30.343 (SE: 4.198)	< 0.001
**P_Response Efficacy WITH**		
P_Self Efficacy	0.458 (SE: 0.101)	< 0.001

^a^*P* Perceived

**Fig. 2:**
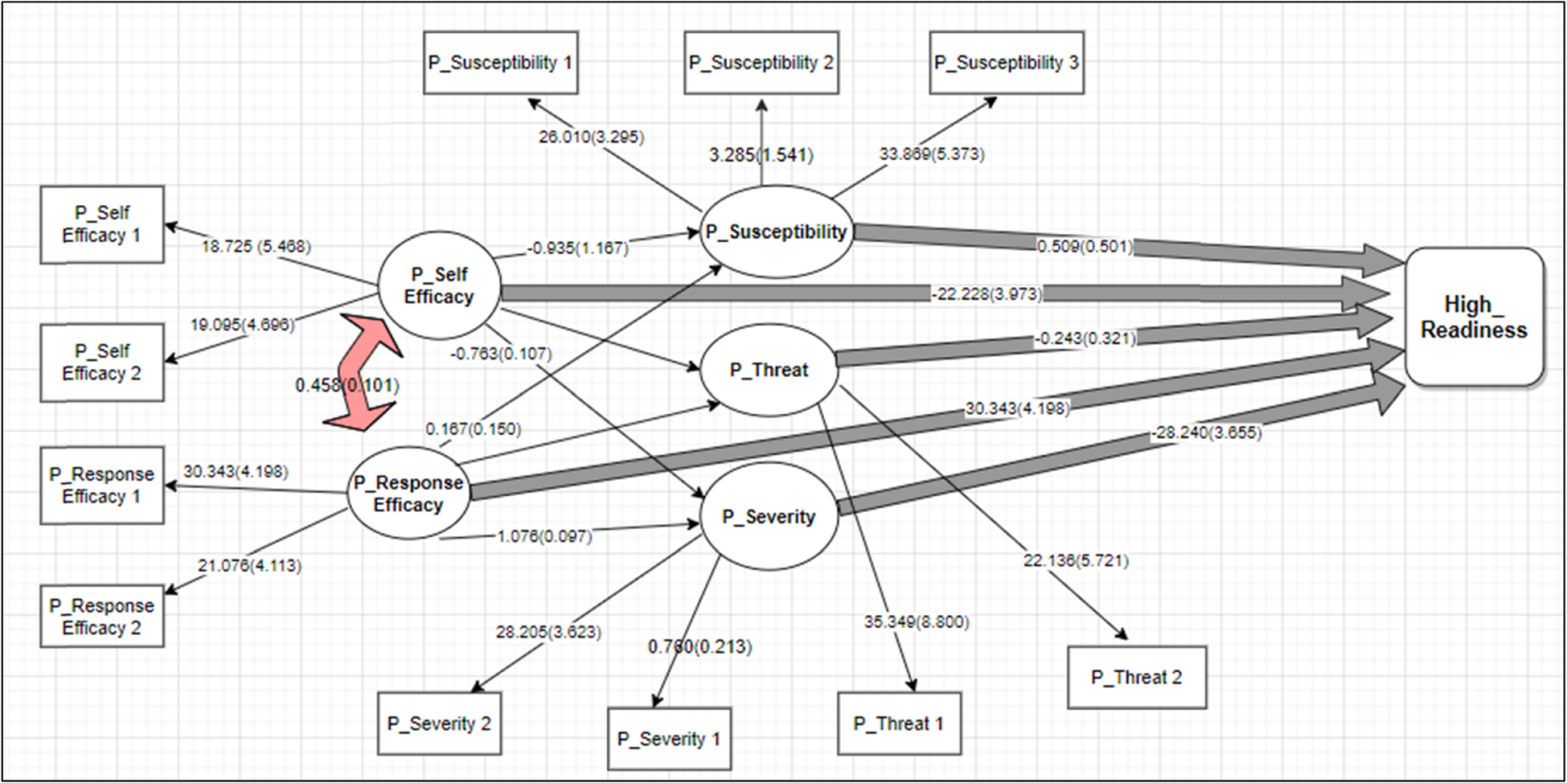
The relationship among EPPM constructs reflected on the SEM. (*P: Perceived)

Findings showed a significant relationship between the Perceived_Susceptibility and P_Response Efficacy; between P_Susceptibility and P_Self Efficacy, P_Severity, and P_Response Efficacy; between P_Severity and P_Self Efficacy. There was also a significant relationship between P_Threat and P_Response Efficacy; between P_Threat and P_Self Efficacy. The relationship between High_Readiness and P_Self Efficacy, between High_Readiness and P_Severity; High_Readiness and P_Response Efficacy also was significant. The relationship between P_Response Efficacy and P_Self Efficacy also was significant (All *P* < 0.05). However, the relationships between High_Readiness and P_Threat; and High_Readiness and P_Susceptibility were not significant (Both *P* > 0.05). For more information, please refer to Fig. 2.

## Discussion

The present study examines the EPPM in which an additive relationship exists between perceived severity and susceptibility (i.e., threat) and between perceived response and self-efficacy to quit (i.e., efficacy) to determine the model’s predictive validity regarding the model’s effectiveness on readiness to quit smoking and lung cancer risk perception. Risk communication is about creating and promoting awareness of cancer-related health risks. The EPPM contains both of these components and has been effectively used in various health topics in the past [44] but has not been examined in the context of lung cancer risk perception and intention to quit. Although the relationship between risk perception and health behavior, in general, has extensively been researched; the association between lung cancer risk perception and readiness to quit is unclear [45].

Studies have suggested causal relationships between lung cancer risk perception and cigarette-smoking behaviors in in different directions (positive, negative, and not significant) [46, 47]. Moreover, instead of causality relations, some studies have simply reported a positive correlation between lung cancer risk perception and smoking behaviors [48, 49], whereas other studies have reorted different associations [50, 51]. Perceived lung cancer risk influences people to take action to lower their risk of getting lung cancer [52]. Intention to quit smoking has been chosen as a behavior change that can reduce smoker’s chance to contract lung cancer [53]. Stopping smoking at any age could lead to decreasing the risk of lung cancer [7]. The results of a study conducted by Ziebarth show that smokers who self-report that they do not plan to quit, are significantly more likely to underestimate their lifetime risk ofdeveloping lung cancer [54, 55].

### Perceived susceptibility

This study showed a significant relationship between Perceived_Susceptibility and both Perceived_Response Efficacy, and PerceivedSelf Efficacy. The significant relationship between Perceived_Susceptibility and Perceived_Response Efficacy indicated that individuals who view themselves at higher risk of developing lung cancer, also believe that quitting smoking can lower this risk (Perceived _Response Efficacy). Moreover, the majority of the participants viewed themselves as capable of quitting smoking (Perceived_Response Efficacy). Most of the participants (199) smoked 1–2 packs per day and only 16 of them smoked 2–4 packs per day (heavy smokers). It is discussed by many researchers that heavy smokers are more likely to underestimate their true risk of developing lung cancer [55, 56]. Therefore, we can assume that most of our participants (non-heavy smokers) had a higher perception of their lung cancer risk. However, we can’t make any assumptions about their intention to quit and their quitting process which is similar to the finding of the study conducted by Poggiolini et al., that even self-efficacy has desirable effects, but that might also serve as a justification to continue smoking instead of stopping smoking [57].

### Perceived severity

There was also a significant relationship between Pperceived_Severity and both Perceived_Response Efficacy, and Perceived_Self Efficacy. The relationship between Perceived_Severity and Perceived_Response Efficacy indicated that individuals who think they will have low lung cancer survival, also positively believed that quitting smoking can improve this outcome. In addition, when the perceived severity is high, it has a positive effect on perceived self-efficacy. This means that even if they are aware of the long-term consequences of smoking, they also view themselves as capable of quitting smoking. Such results give us valuable information about the likelihood of our population participating in smoking cessation programs. We may presume that they might have never had any chance to participate in such educational programs. Self-efficacy is usually associated with self-control and self-regulation, which is extremely critical in adapting the target behavior [18]. Smokers who are not in the status to quit smoking (low readiness to quit) are considerably more inclined to underestimate this mortality risk [54] which is similar to our results that there was a significant correlation between Perceived_Severity and Perceived_Self Efficacy. Another explanation for this finding is based on the cognitive dissonance theory [58]. Because it is not easy to quit smoking, most smokers (in our population, people with low readiness to quit) decide to change their beliefs rather than their behaviors. In this status, they underestimate the health risks of smoking and they focus more on positive aspects of smoking (e.g., increasing concentration).

### Perceived threat

The next significant relationship was between Perceived_Threat and both Perceived_Response Efficacy and Perceived_Self Efficacy. When both threat and efficacy appraisals are high, the individual will enter a cognitive process to control the danger rather than the fear. In this stage, people are more likely to engage in adaptive behavior which is taking actual steps such as quitting smoking [59, 60]. Therefore, we could suggest that educational programs for smoking cessation in a similar population could be more focused on increasing the Perceived_Threat of the disease. This also could be explained by cognitive dissonance theory. Smokers who feel distressed by knowing the long-term health risks of smoking and also are capable of taking actual steps to quit, in fact, are lowering their dissonance by changing their behavior [58].

### High readiness

However, the relationships between the High_Readiness with two EPPM theoretical variables (Percived_Threat, and Percived_Susceptibility) were not significant. Wong and Cappella have concluded in their study that Percived_Threat is more important for smokers with Low_Readiness, whereas efficacy is most important among smokers with High_Readiness [14]. This is also in line with our results that there was no significant association between Perceived_Threat and intention to quit among smokers with High_Readiness. It could be explained that smokers’ high readiness to quit is not necessarily related to their higher Perceived_Threat or Perceived_Susceptibility of lung cancer. In other words, their high intention to quit is not an indicator that they view themselves at a higher risk of developing lung cancer. Based on our final results, it may be they are more inclined to quit, because of other factors such as other chronic diseases (e.g. heart disease, diabetes, Chronic Obstructive Pulmonary Disease, etc.), respecting family members or partner’s request to quit, cigarette’s bad smell, and reducing the financial burden of smoking.

The limitations of this study must be acknowledged. Recruiting female smokers was challenging because this topic is sensitive among female participants in Iranian culture. Another limitation was the unbalanced sample size in the subgroups which may make the statistical tests under powerand therefore, the results of the study could be nonsignificant. The lack of a control group to compare the perceived risk between smokers and non-smokers,gathering responses through a self-report system, and the cross-sectional nature of this study could be other limitations of the present study. Finally, theinconsistent findings in the literature about the relationship between lung cancer risk perception and smoking behaviors (e.g.,readiness to quit) could impact interpreation of our results. Designing future longitudinal studies to further investigate this association in our population is suggested.

## Conclusion

Both threat and efficacy were important for smokers with low readiness to quit, whereas efficacy was most important among smokers with high readiness to quit. Our findings provided practical support for the utility of the Extended Parallel Process Model in designing effective educational programs to promote lung cancer awareness. The study findings could be extremely helpful in developing public health interventions to quit smoking based on smokers’ stage of change and readiness to quit.

## Data Availability

The datasets used and analyzed for this current study are available from the first author and/or the corresponding author on reasonable request.

## References

[1] Ghasemian A, Rezaei N, Saeedi Moghaddam S (2015). Tobacco Smoking Status and the Contribution to Burden of Diseases in Iran, 1990–2010: findings from the Global Burden of Disease Study 2010. Arch Iran Med.

[2] Rezaei S, Akbari Sari A, Arab M, Majdzadeh R, Mohammadpoorasl A. Estimating the economic burden of cancer deaths attributable to smoking in Iran. J Res Health Sci. 2015;15(4):228-33. 26728908

[3] Moosazadeh M, Ziaaddini H, Mirzazadeh A, Ashrafi-Asgarabad A, Haghdoost AA. Meta-analysis of Smoking Prevalence in Iran. Addict Health. 2013 Summer-Autumn;5(3-4):140-53. PMC3905476 24494171

[4] Lung Cancer Statistics | How Common is Lung Cancer. Accessed December 30, 2019. https://www.cancer.org/cancer/lung-cancer/about/key-statistics.html

[5] Cancer of the Lung and Bronchus - SEER Stat Fact Sheets. Accessed December 3, 2016. https://seer.cancer.gov/statfacts/html/lungb.html

[6] O'Keeffe LM, Taylor G, Huxley RR, Mitchell P, Woodward M, Peters SAE. Smoking as a risk factor for lung cancer in women and men: a systematic review and meta-analysis. BMJ Open. 2018;8(10):e021611. 10.1136/bmjopen-2018-021611. 10.1136/bmjopen-2018-021611 PMC6194454 30287668

[7] Alberg AJ, Nonemaker J (2008). Who is at high risk for lung cancer? Population-level and individual-level perspectives. Semin Respir Crit Care Med.

[8] Alberg AJ, Samet JM (2003). Epidemiology of lung cancer. Chest..

[9] CDC-Division of Cancer Prevention and Control C. CDC - What Are the Risk Factors for Lung Cancer? Smoking. Published 2019. Accessed 3 Dec 2018. https://www.cdc.gov/cancer/lung/basic_info/risk_factors.htm

[10] Briss P, Rimer B, Reilley B, Coates RC, Lee NC, Mullen P, Corso P, Hutchinson AB, Hiatt R, Kerner J, George P, White C, Gandhi N, Saraiya M, Breslow R, Isham G, Teutsch SM, Hinman AR, Lawrence R, Task Force on Community Preventive Services (2004). Promoting informed decisions about cancer screening in communities and healthcare systems. Am J Prev Med.

[11] Witte K, Gary Meyers, Martel D. SAGE Books - Effective Health Risk Messages: A Step-by-Step Guide. Published 2001. Accessed 31 Dec 2019. http://sk.sagepub.com/books/effective-health-risk-messages

[12] Chen LS, Kaphingst KA (2010). Risk perceptions and family history of lung Cancer: differences by smoking status. Public Health Genomics.

[13] Witte K (1994). Fear control and danger control: a test of the extended parallel process model (EPPM). Commun Monogr.

[14] Wong NCH, Cappella JN (2009). Antismoking threat and efficacy appeals: effects on smoking cessation intentions for smokers with low and high readiness to quit. J Appl Commun Res JACR.

[15] Morman MT (2000). The influence of fear appeals, message design, and masculinity on men’s motivation to perform the testicular self-exam. J Appl Commun Res.

[16] Hubbell AP (2006). Mexican American women in a rural area and barriers to their ability to enact protective behaviors against breast cancer. Health Commun.

[17] Cho H, Salmon CT (2006). Fear appeals for individuals in different stages of change: intended and unintended effects and implications on public health campaigns. Health Commun.

[18] Birmingham WC, Hung M, Boonyasiriwat W, Kohlmann W, Walters ST, Burt RW, Stroup AM, Edwards SL, Schwartz MD, Lowery JT, Hill DA, Wiggins CL, Higginbotham JC, Tang P, Hon SD, Franklin JD, Vernon S, Kinney AY (2015). Effectiveness of the extended parallel process model in promoting colorectal cancer screening: EPPM in CRC screening. Psychooncology.

[19] Brown VJ (2014). Risk perception: It’s personal. Environ Health Perspect.

[20] Fischhoff B, Bostrom A, Jacobs Quadrel M (1993). Risk Perception and Communication. Vol 14.

[21] Termeh Zonouzy V, Niknami S, Ghofranipour F, Montazeri A (2018). An educational intervention based on the extended parallel process model to improve attitude, behavioral intention, and early breast cancer diagnosis: a randomized trial. Int J Women’s Health.

[22] Chen L, Yang X, Fu L, Liu X, Yuan C. Using the Extended Parallel Process Model to Examine the Nature and Impact of Breast Cancer Prevention Information on Mobile-Based Social Media: Content Analysis. JMIR MHealth UHealth. 2019;7(6). 10.2196/13987. 10.2196/13987 PMC6613324 31237239

[23] Ooms J, Jansen C, Hoeks J (2015). The EPPM put to the test: evaluating four basic propositions. Dutch J Appl Linguist.

[24] Chen L, Yang X (2019). Using EPPM to evaluate the effectiveness of fear appeal messages across different media outlets to increase the intention of breast self-examination among Chinese women. Health Commun.

[25] Mazloomy Mahmoodabad SS, Gerayllo S, Mizani N. Factors Influencing Skin Cancer Preventive Behaviors Based on the Extended Parallel Process Model in Yazd University of Medical Sciences Students, 2017. J Community Health Res. 2019. Published online October 12, 2019. 10.18502/jchr.v8i3.1563.

[26] (PDF) The Effects of Fear Appeals and Message Format on Promoting Skin Cancer Prevention Behaviors among College Students. Accessed 28 Mar 2020. https://www.researchgate.net/publication/339450046_The_Effects_of_Fear_Appeals_and_Message_Format_on_Promoting_Skin_Cancer_Prevention_Behaviors_among_College_Students

[27] Pengchit W, Walters ST, Simmons RG, Kohlmann W, Burt RW, Schwartz MD, Kinney AY (2011). Motivation-based intervention to promote colonoscopy screening: an integration of a fear management model and motivational interviewing. J Health Psychol.

[28] Gollust SE, Schroeder SA, Warner KE (2008). Helping smokers quit: understanding the barriers to utilization of smoking cessation services. Milbank Q.

[29] Sharifirad G, charkazi A, Berdi-Ghourchaei A, Shahnazi H, Moudi M, et al. Smoking Behavior Based on Stages of Change Model Among Iranian Male Students in 2009-2010 Academic Year. Zahedan J Res Med Sci. 2012;14(1):e93610.

[30] Jafari T, Shahrokhi S. Prevention, Treatment, and Control of Cigarett Use. Published online 2010. http://nafass.old.piho.ir/UploadedFiles/XFiles/nafass/pdf/dastor%20ol%20amal%20ejrayee.pdf

[31] Soltanipour S, Heidarzadeh A, Jafarinezhad A (2014). Reliability and validity of the Persian [Farsi] version of the risk perception survey-diabetes mellitus. East Mediterr Health J.

[32] Graybill FA (1961). An Introduction to Linear Statistical Models, Vol 1.

[33] Guenther WC (1977). Desk calculation of probabilities for the distribution of the sample correlation coefficient. Am Stat.

[34] Zar JH. Biostatistical analysis. 2nd. Prentice-Hall - Google Search. Biostatistical Analysis. Published 1984. Accessed 6 Nov 2019. https://www.google.com/search?q=Biostatistical+Analysis.+Second+Edition.+Prentice-Hall&oq=Biostatistical+Analysis.+Second+Edition.+Prentice-Hall&aqs=chrome..69i57.500j0j7&sourceid=chrome&ie=UTF-8

[35] Tinsley H, Brown S. Handbook of Applied Multivariate Statistics and Mathematical Modeling. 1st ed: Academic Press; 2000. 10.1016/B978-0-12-691360-6.X5000-9.

[36] Ryan H, Trosclair A, Gfroerer J. Adult current smoking: differences in definitions and prevalence estimates—NHIS and NSDUH, 2008. J Environ Public Health. 10.1155/2012/918368. 10.1155/2012/918368 PMC3357540 22649464

[37] Tindle HA, Stevenson Duncan M, Greevy RA (2018). Lifetime Smoking History and Risk of Lung Cancer: Results From the Framingham Heart Study. JNCI J Natl Cancer Inst.

[38] Wehring HJ, Liu F, McMahon RP, Mackowick KM, Love RC, Dixon L, Kelly DL (2012). Clinical characteristics of heavy and non-heavy smokers with schizophrenia. Schizophr Res.

[39] Key Statistics for Lung Cancer. Published 2019. Accessed 3 Dec 2018. https://www.cancer.org/cancer/non-small-cell-lung-cancer/about/key-statistics.html

[40] Peto J (2012). That the effects of smoking should be measured in pack-years: misconceptions 4. Br J Cancer.

[41] Prochaska JO, Diclemente CC, Norcross JC (1992). In search of how people change. Applications to addictive behaviors. Am Psychol.

[42] Jhun H-J, Seo H-G (2006). The stages of change in smoking cessation in a representative sample of Korean adult smokers. J Korean Med Sci.

[43] Muthén LK, Muthén B. Mplus statistical analysis with latent variables: User’s guide. Muthén & Muthén; 2010. Accessed 31 Dec 2019. http://www.statmodel.com

[44] Gore TD, Bracken CC (2005). Testing the theoretical design of a health risk message: reexamining the major tenets of the extended parallel process model. Health Educ Behav Off Publ Soc Public Health Educ.

[45] Chen L-S, Kaphingst KA, Tseng T-S, Zhao S (2016). How are lung cancer risk perceptions and cigarette smoking related?—testing an accuracy hypothesis. Transl Cancer Res.

[46] Lundborg P, Lindgren B (2004). Do they know what they are doing? Risk perceptions and smoking behaviour among Swedish teenagers. J Risk Uncertain.

[47] Finney Rutten LJ, Blake KD, Hesse BW, Augustson EM, Evans S (2011). Illness representations of lung cancer, lung cancer worry, and perceptions of risk by smoking status. J Cancer Educ Off J Am Assoc Cancer Educ.

[48] Weinstein ND, Marcus SE, Moser RP (2005). Smokers’ unrealistic optimism about their risk. Tob Control.

[49] Helweg-Larsen M, Nielsen GA (2009). Smoking cross-culturally: risk perceptions among young adults in Denmark and the United States. Psychol Health.

[50] Liu J-T, Hsieh C-R (1995). Risk perception and smoking behavior: empirical evidence from Taiwan. J Risk Uncertain.

[51] Helweg-Larsen M, Stancioff LM (2008). Acculturation matters: risk perceptions of smoking among Bosnian refugees living in the United States. J Immigr Minor Health.

[52] Katapodi MC, Lee KA, Facione NC, Dodd MJ (2004). Predictors of perceived breast cancer risk and the relation between perceived risk and breast cancer screening: a meta-analytic review. Prev Med.

[53] Savoy E, Reitzel LR, Scheuermann TS, Agarwal M, Mathur C, Choi WS, Ahluwalia JS (2014). Risk perception and intention to quit among a tri-ethnic sample of nondaily, light daily, and moderate/heavy daily smokers. Addict Behav.

[54] Ziebarth NR (2018). Lung cancer risk perception biases. Prev Med.

[55] Ziebarth N (2018). Biased lung Cancer risk perceptions: smokers are misinformed. Jahrb Für Natl Stat.

[56] Masiero M, Lucchiari C, Pravettoni G (2015). Personal fable: optimistic Bias in cigarette smokers. Int J High Risk Behav Addict.

[57] Poggiolini C. High self-efficacy regarding smoking cessation may weaken the intention to quit smoking. Cogent Psychol. 2019;6(1). 10.1080/23311908.2019.1574096.

[58] Fotuhi O, Fong GT, Zanna MP, Borland R, Yong H-H, Cummings KM (2013). Patterns of cognitive dissonance-reducing beliefs among smokers: a longitudinal analysis from the international tobacco control (ITC) four country survey. Tob Control.

[59] Popova L (2012). The extended parallel process model: illuminating the gaps in research. Health Educ Behav.

[60] Allahverdipour H, MacIntyre R, Hidarnia A (2007). Assessing protective factors against drug abuse among high school students: self-control and the extended parallel process model. J Addict Nurs.

